# Therapeutics

**Published:** 1878-07

**Authors:** 


### THERAPEUTICS.

JABORANDI.—*Pilocarpus Pinnatus* is a drug which invites further study. After the researches of Dr. Ringer and Mr. Martindale, an English pharmacist, there can be no doubt of the powerful diaphoretic and sialogogue properties possessed by it. The secret of its mighty diaphoretic and sialogogue strength undoubtedly exists in its alkaloid, recently discovered by Mr. Gerard, and the most efficient salt of this alkaloid is generally believed to be the nitrate; its price, however, effectually excludes it at present from general use. The price asked for it in New York is about $25 per drachm; but as better and easier methods of eliminating it are discovered, the price will correspondingly decline.

Half a grain of the nitrate is said to produce the effect of a full dose of jaborandi. One drop of the solution of the nitrate (1 grain to 1 ounce) put into the eye will contract the pupil to the size of a pin's head. From a report of some interesting physiological experiments performed on a dog and a rabbit at University College, London, we find that $\frac{1}{10}$ of a grain of the alkaloid produced profuse salivation, which was readily checked by administering $\frac{1}{200}$ of a grain of sulphate of atropine. Mr. Gerard thinks that the best pharmaceutical preparation is the fluid extract. In this city jaborandi has been used with success in one drachm doses, infused in a cup of boiling water, and the whole drunk (without being strained). In a short while it produced an excessive flow of saliva followed by profuse diaphoresis. No nausea followed in the two cases reported by the physician for whom the writer prepared the remedy.

Treatment of Boils by Arnica.—Dr. N. Planat has adopted (*Journal de Thérapeutique*) the use of arnica in all cases of superficial acute inflammation, as boils, angina, erysipelas, etc. He states that arnica cuts short all furuncular eruptions, except those accompanied by diabetes, with remarkable promptness. For external use, he employs a mixture of extract of fresh arnica flowers, ten parts; honey, twenty parts. If this be too liquid, he adds lycopodium. The mixture is applied to the inflamed part and covered with oiled silk. Equally good results will be obtained in the same cases by the internal administration of tincture of arnica in doses of twenty-five to thirty drops every two hours. M. Planat adds that the extinction of the furuncular eruption is so rapid that it seems impossible to deny a specific elective action.

Vaseline as Excipient. (*Journal de Médecine et de Chirurgie pratique.*)—Vaseline is an excellent excipient for: potassium iodide, tannin, chloral hydrate, chloroform, camphor, morphine, atropine, iodine and phosphorus. The vaseline does not become rancid and the alkaloids do not decompose.

As it contains no fat, it cannot be used where chemical combination is desired to form oleates.

